# Neuronal induction and bioenergetics characterization of human forearm adipose stem cells from Parkinson’s disease patients and healthy controls

**DOI:** 10.1371/journal.pone.0265256

**Published:** 2022-03-15

**Authors:** Ingrid González-Casacuberta, Dolores Vilas, Claustre Pont-Sunyer, Ester Tobías, Judith Cantó-Santos, Laura Valls-Roca, Francesc Josep García-García, Glòria Garrabou, Josep Maria Grau-Junyent, Maria Josep Martí, Francesc Cardellach, Constanza Morén

**Affiliations:** 1 Cellex-Institut d’Investigacions Biomèdiques August Pi i Sunyer (IDIBAPS), Faculty of Medicine and Health Science, University of Barcelona, Spain; 2 Internal Medicine Department, Hospital Clínic of Barcelona, Barcelona, Spain; 3 Biomedical Research Networking Center on Rare Diseases (CIBERER), Madrid, Spain; 4 Neurodegenerative Diseases Unit, Neurology Service, University Hospital Germans Trias i Pujol, Badalona, Catalonia, Spain; 5 Neurology Unit, Hospital General de Granollers, Universitat Internacional de Catalunya, Barcelona, Catalonia, Spain; 6 Movement Disorders Unit, Neurology Service, Institut de Neurociències, University of Barcelona, Hospital Clínic de Barcelona, Barcelona, Catalonia, Spain; 7 Centro de Investigación Biomédica en Red sobre Enfermedades Neurodegenerativas (CIBERNED: CB06/05/0018-ISCIII), Barcelona, Catalonia, Spain; Università degli Studi della Campania, ITALY

## Abstract

Neurodegenerative diseases, such as Parkinson’s disease, are heterogeneous disorders with a multifactorial nature involving impaired bioenergetics. Stem-regenerative medicine and bioenergetics have been proposed as promising therapeutic targets in the neurologic field. The rationale of the present study was to assess the potential of human-derived adipose stem cells (hASCs) to transdifferentiate into neuronal-like cells (NhASCs and neurospheres) and explore the hASC bioenergetic profile. hASC neuronal transdifferentiation was performed through neurobasal media and differentiation factor exposure. High resolution respirometry was assessed. Increased MAP-2 neuronal marker protein expression upon neuronal induction (p<0.05 undifferentiated hASCs vs. 28–36 days of differentiation) and increased bIII-tubulin neuronal marker protein expression upon neuronal induction (p<0.05 undifferentiated hASCs vs. 6-28-36 days of differentiation) were found. The bioenergetic profile was detectable through high-resolution respirometry approaches in hASCs but did not lead to differential oxidative capacity rates in healthy or clinically diagnosed PD-hASCs. We confirmed the capability of transdifferentiation to the neuronal-like profile of hASCs derived from the forearms of human subjects and characterized the bioenergetic profile. Suboptimal maximal respiratory capacity trends in PD were found. Neuronal induction leading to positive neuronal protein expression markers is a relevant issue that encourages the suitability of NhASC models in neurodegeneration.

## Introduction

Neurodegenerative diseases, including Parkinson’s disease (PD), Alzheimer’s disease, or amyotrophic lateral sclerosis (ALS), are heterogeneous disorders with multifactorial pathogenesis and etiologies. Their prevalence is increasing with the rise of global population and lifespan. Even though many therapeutic approaches have been tested, there are currently no effective preventive or precise treatment options. The multifactorial nature of such disorders includes different molecular pathways related to impaired bioenergetics. Indeed, mitochondrial dysfunction, directly implicated in cell bioenergetics, is emerging as a key feature in the etiopathogenesis of these age-related neurodegenerative diseases. Previous findings of our group demonstrated suboptimal bioenergetic profiles in fibroblast models derived from different genetic forms of PD patients [1–3].

Adult stem cells are multipotent and undifferentiated cells present in some tissues of adult organisms. These cells present self-renewal capacity and differentiate into specialized cells. Thus, they may represent a promising implement for replacement cell therapy purposes in a panoply of diseases and processes, including neurodegeneration. Evidence underpinning the potential of stem cell approaches in the neuropathological context is continuously emerging in the recent literature, such as the finding that dental pulp stem cells stimulate neuronal differentiation of PC12 cells [4].

Adult stem cells are also considered a highly encouraging tool for regenerative medicine [5]. Adipose tissue is the most accessible and abundant source of adult stem cells. The relatively minimally invasiveness at collection, the ability of adult stem cells to differentiate into different cell lineages, and their safety during autologous transplantation allow adipose stem cells to be an alternative origin for bone marrow cells [6]. Adipose tissue consists of mature adipocytes and a heterogeneous cell population, such as the stromal vascular fraction [7]. This vascular fraction comprises various cell types, including immune cells, fibroblasts, pericytes, endothelial cells, and adipogenic progenitor stromal cells, that attach to collagen fibers [5]. In culture, human adipose-derived stem cells (hASCs) express cell surface markers similar to those expressed by mesenchymal stem cells, including SH3, CD44, CD90 and CD105. However, they do not express the hematopoietic marker CD45 and the endothelial marker CD31 [8, 9]. As adipose tissue is comprised of heterogeneous population of partially differentiated cells of adipocyte lineage, angiogenesis, neovascularization and tissue repair, could be closely related to the hASCs function in a complex relationship [10]. Due to such interactions and heterogeneous features within adipose tissue, specific biological mechanisms involved in adequate hASC functioning and homeostasis, including stem pluripotency/transdifferentiation capacities and bioenergetic fingerprints, remain unknown. Interestingly, both stem [11] and bioenergetic [12] tools, explored in the present study, are considered as promising therapeutic novel targets in the neurologic field. However, recent studies have shown that adipose derived stem cells may promote tumour progression so caution should be taken into account when conducting autologous fat grafts [13].

Epigenesis could be related to cellular differentiation during developmental stages and is controlled by different factors (growth and environmental factors, among others) [14]. The genetic reprogramming process involves these factors directly or indirectly in the genetic transcription and expression [15]. The transdifferentiation mechanism could induce adipose-derived stem cells into neural and glial phenotypes [11].

Previous findings demonstrate the relevance of adipose tissue nervous regulation on metabolic and secretory activities, as well as apoptosis, proliferation, differentiation and transdifferentiation [16, 17]. The neuronal feedback cycle between the brain and adipose tissue is essential for energetic homeostasis. The respectively oxidative and lipolytic metabolic nature of neuronal and adipose tissue, confers a higher level of complexity in their relationship and molecular studies carried out in both types of biological sources and derived cell models, especially those related to bioenergetics. Although little is known about hASCs bioenergetic profile, a recent first approach to hASC bioenergetics demonstrated that these stem cells could affect metabolic homeostasis by promoting damaged mitochondrial clearance through mitophagy, therefore delaying aging [18].

The rationale of the present study is to assess the potential of hASCs to transdifferentiate into neuronal-like cells (NhASCs and neurospheres), together with the assessment of their bioenergetic fingerprint. For this purpose, hASCs derived from both healthy controls and clinically diagnosed PD (*PD*) patients were included in this study.

The present work contributes to the characterization of a stem cell model and its bioenergetic profile as a potential valid novel target for treating neurodegenerative conditions.

## Materials and methods

### Subjects and sample collection

Samples from healthy controls and patients with PD (n = 3) were used in this study; written informed consent was obtained before subcutaneous fat was collected. All methods were performed in accordance with the relevant guidelines and regulations following the Declaration of Helsinki. The protocols used for the collection were approved by the Hospital Clínic of Barcelona Ethics Committee (HCB EC number 2018/0228), *comité de ética para investigación con medicamentos*, *CEIm*. Skin-punch biopsies with subcutaneous adipose tissue (4 mm) were obtained from participants’ forearms. Subcutaneous adipose tissues were separated from the skin and used for cell isolation. Human subcutaneous adipose tissues were washed with growth medium and dissected into small pieces (less than 0.2 cm diameter). For example, from a given biopsy, 18 pieces could be obtained.

### Adipose cell culture and maintenance

The small pieces of adipose tissues were explanted in sterile 10 cc dishes. A sterilized coverslip was used to cover the tissue to prevent tissue floating in the medium. Complete media for hASCs was prepared as follows: Dulbecco’s Modified Eagle Medium (DMEM) supplemented with 10% fetal bovine serum (FBS), 1% nonessential amino acids (NEAAs), 1% pyruvate and 0.5% penicillin-streptomycin antibiotics. Media were changed every 3 days until migrated cells reached 70–80% confluency. The cells were dissociated from the well with Accutase (Millipore) and seeded at a ratio of 1:3 in ordinary culture dishes with growth medium. Passage was conducted as follows: medium was removed; cells were washed with sterile 1x PBS and incubated with Accutase (1 mL/dish; 100 x 20 mm) at 37°C for 3–5 min. Growth medium was added to the dissociated cells. The medium containing cells was splitted in three individual dishes. Cells were cultured and maintained in growth medium at 37°C with 5% CO_2_ and 95% air.

### Neurosphere hASC transdifferentiation

When 70%–80% confluency was reached, hASCs were collected and pelleted(2000 g, 10 min). Cell pellets were resuspended in neurosphere media, transferred to an uncoated 12-well plate for further maintenance and experiments (37°C with 5% CO_2_ 95% air). Cells were cultured in DMEM-F12 (1:1) containing: 1x B27 (Thermo Fisher catalog n° #17504–044), 20 μL leukemia inhibitor (10 ng/mL), essential fibroblast growth factor (40 ng/mL FGF2 or bFGF, catalog n° #233-FB-025), epidermal growth factor (10 ng/mL EGF) and 1% penicillin-streptomycin antibiotics were harvested in sterile 6-well plates. The neurospheres were cultured by changing the media every 5 days.

### Neuron-like hASC transdifferentiation (NhASC)

On day 1, adipose stem cells were seeded at 8000 cells/ml density, and 3 ml were harvested on a 6-well plate previously coated with fibronectin (Sigma #F1141), ~1 mg/ml. On day 2, h-ASC media was removed and replaced with differentiation neurobasal medium (Thermo Fisher catalog no #21103–049) containing B-27 (Thermo Fisher catalog no #17504–044), 250 ng/ml human sonic Hedgehog (sHH, catalog no #1845-SH-025), 100 ng/ml human fibroblast growth factor 8 (FGF8, catalog no #423-F8-025), 50 ng/ml human basic fibroblast growth factor (bFGF, catalog no #233-FB-025) and antibiotic/antimycotic solution (Sigma catalog no #A5955). On day 12, 1 ml of medium was aspirated, and 2 ml of differentiation medium containing 50 ng/ml human BDNF (catalog number #248-BD-025) was added. This last step was repeated every 8 days (on days 20, 28, 36 and 40), and NhASCs were maintained until a maximum of 42–46 days.

### Flow cytometry

Cells were harvested, washed and centrifuged for 5 min at 300 g. Pellets were resuspended in sterile complete media, and cell counts were determined. Approximately 500,000 cells were used for each reaction, including different cell types for comparison purposes (fibroblasts and hASCs). Cells were recorded on a BD LSR II flow cytometer (BD Biosciences, Oxford, UK) using BD FACSDiva software, and data were analyzed using FlowJo software (TreeStar., Ashland, OR, USA) [19]. The following panel of antibodes were used for hASC characterization: APC mouse anti-human CD45 (BD Biosciences catalog no #555485), PE mouse anti-human CD34 (BD Horizon, BD Biosciences, catalog no #562577), PE mouse anti-human CD73 (BD Biosciences catalog no #550257), PE mouse anti-human CD90 (Biosciences catalog no #555596) and PE mouse anti-human CD-105 (BD Biosciences catalog no #560839). The percentage of fluorescence in hASCs was determined.

### Western blot analysis and densitometry

Western blot experiments were conducted as reported elsewhere [20]. Briefly, hASCs and NhASCs were lysed and placed on ice for 15 min with RIPA buffer supplemented with a 1x Halt protease inhibitor cocktail (Pierce). Lysates were centrifuged (21,000 g, 4°C, 5 min), and soluble material was used for western blot analysis. Protein levels were assessed by using bicinchoninic acid (BCA) kit (Pierce Thermo Fisher; Basingstoke, UK) as described previously [21]. 20–40 μg protein from the soluble material were resolved under reducing conditions in either NuPAGE 4–12% polyacrylamide gels (Invitrogen, Carlsbad, CA, USA) with 2 (N morpholino) ethane sulfonic acid buffer or NuPAGE 12% (Invitrogen, Carlsbad, CA, USA) with MOPS buffer. Proteins were transferred onto Immobilon polyvinylidene difluoride or nitrocellulose membranes (Millipore, Watford, UK). Blocking was conducted with 10% skimmed milk powder diluted in PBS. Primary antibodies detecting microtubule-associated protein 2 (MAP2) (1:1000 MAP2, Invitrogen), βIII tubulin (1:1000 ab7751 Abcam), and β actin (1:10000, Sigma) were used. Secondary antibodies conjugated with horseradish peroxidase against mouse and rabbit IgG were used (1:2000) (Dako, Glostrup, Denmark). ECL reagent (GE Healthcare, Bucks, UK) was used for densitometry detection of the blots and the signals were detected through the LAS4000 system. Band densities were assessed using ImageJ and ImageQuant analysis software.

### High-resolution respirometry studies

Basal respiration, proton leakage, maximal respiration and residual respiration was conducted through high-resolution assessment Oroboros and Seahorse technologies, by assessment of respiration OXPHOS slope states indicating oxygen consumption rates (OCRs).

### Oxidative assessment (Oroboros 2k-oxygraph)

For high-resolution respirometry, we used the two-chamber respirometer Oxygraph-2k (O2k, OroborosTM instruments, Innsbruck, Austria) [22, 23], a barometric transducer that enables oxygen calibrations through the DatLab software. Oxygen consumption by hASC samples suspended in 2.1 mL of MIR05 solution was monitored under 37°C and controlled stirring (300 rpm). We determined the primary respiratory states. First, the routine, or natural basal respiration of intact cells in the absence of substrates and inhibitors. Second, the proton leak (no-phosphorylation), after ATP synthase inhibition by oligomycin (1 μg/mL), enabling the analysis of the natural proton leak across the inner mitochondrial membrane (IMM). Third, the maximal respiratory capacity, or ETS, in the presence of the ionophore and mitochondrial uncoupler carbonyl cyanide m-chlorophenylhydrazone (CCCP, 1 or 2 μM), which directly transports protons across the IMM, without the intervention of the ATP synthase proton channel, subsequently leading to rapid consumption of oxygen lacking of ATP generation. Finally, residual respiration, or Rox, was captured after complex III inhibition by antimycin A (AA, 3 μM) [24]. After the oxygen consumption analysis, the entire reaction mixture was removed from the O2k chamber and used for total protein assessment through the protein BCA method [21], leading to pmol/min.ug protein units.

### Oxidative assessment (Agilent Seahorse instrument XFe24 Analyzer)

OCRs were assessed in intact adherent hASCs using an Agilent Seahorse TM XFe24 Analyzer (Seahorse Bioscience, Wilmington, DE, USA) and the Cell Mito Stress Test [25]. 15,000–30,000 hASCs/per well were seeded in 24-well Seahorse culture plates and maintained overnight in 250 μL of growth medium to allow adhesion. Each cell line and condition were seeded in triplicates. After 24 hours, growth medium was removed, cells were washed once and Seahorse XF Base Medium (Seahorse Bioscience) was added. Then, plates were incubated according to the manufacturer’s protocol (30 min at 37°C without CO_2_). Oxygen consumption was analyzed both under basal conditions and after adding oligomycin. Similar to previously described CCCP tritiation in Oroboros technology, uncoupler carbonyl cyanide-4-(trifluoromethoxy) phenylhydrazone (FCCP) was added to measure the maximal respiratory capacity. The ionophore FCCP, as well as CCCP, directly transports protons across the IMM, avoiding the ATP synthase proton channel, and leading to rapid consumption of oxygen (with no ATP production). Rotenone and antimycin-A, inhibitors of complex I and III, respectively (Sigma-Aldrich), were added to assess nonspecific nonmitochondrial respiration. Spare respiratory capacity was calculated as the maximal after basal OCR subtraction (%) and basal/maximal respiratory ratio was calculated as the ratio between basal and maximal OCR. Respiration values were normalized to the total cell protein content measured by the BCA assay [21], leading to pmol/min.ug protein units.

## Results

### Characterization of hASCs and their ability to differentiate in neurobasal media

**Fig 1:**
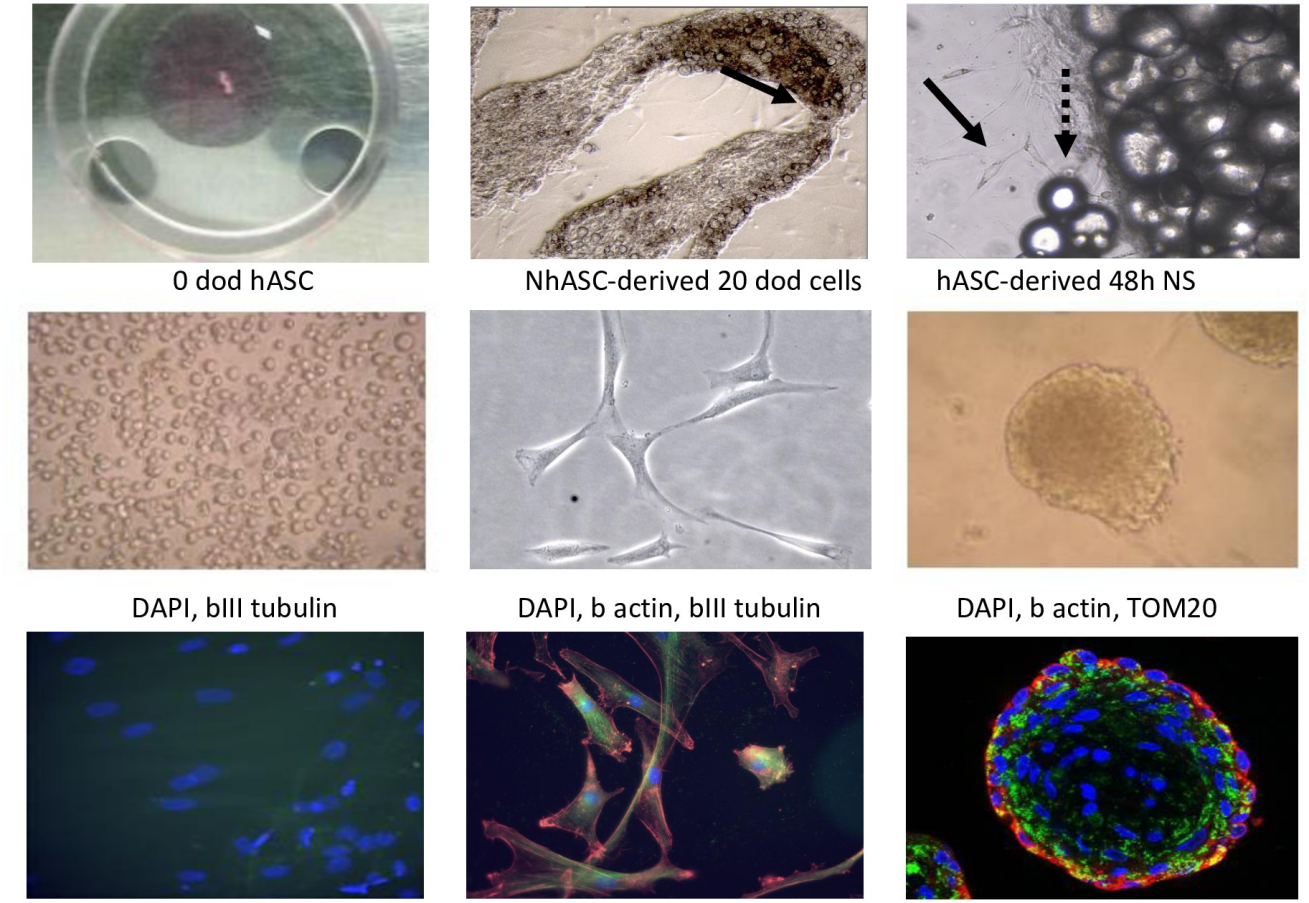
Representative images of hASC and NhASC. Upper panels: From left to right; first panel, forearm adipose tissue biopsy on a Petri dish prior to tissue disgregation; second and third panels, adipose tissue dissected fragments by optical microscopy (5x and 10x, respectively). Adipose tissue and scarce adipose cells generated from tissue explants. Black continuous arrow pointing adipose cells coming out from the explant. Black discontinuous arrow marking typical oil drops from adipose tissue samples. Middle panels: From left to right, first human adipose stem cells at 0h of induction with neurobasal differentiation media; second panel, human derived adipose stem cells after 20 days of differentiation in neurobasal differentiation media; third panel, neurosphere formation after 48 hours of induction. Dod, days of development (differentiation), hASCs, human adipose stem cells, NhASCs, neuronal-like adipose stem cells, NS, neurosphere. Bottom panels: From left to right; first, representative immunofluorescence images of forearm human adipose stem cells undifferentiated at basal timepoint conditions (blue DAPI); second, after 20 days of differentiation (blue DAPI, red b actin, green neuronal bIII tubulin) and third, after neurosphere formation (blue DAPI, red b actin, green TOM20). hASC, human adipose stem cells.

**Fig 2:**
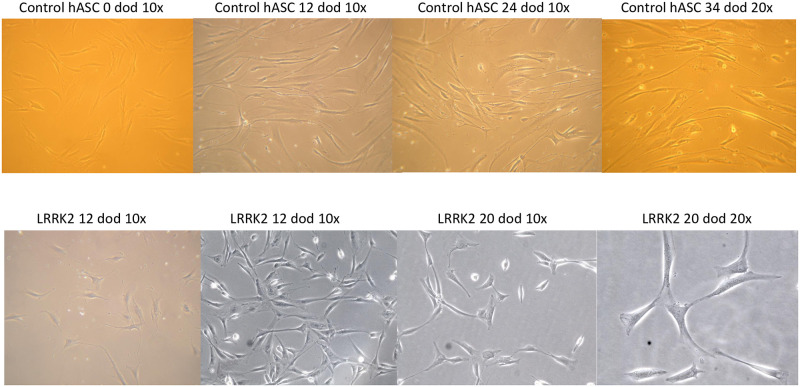
Representative images of hASC. Forearm human adipose stem cells from control and PD-diagnosed lines (upper and lower panels, respectively) over time up to 20 days of differentiation.

Adipose tissue explants led to proliferating hASCs after approximately 3 weeks of biopsy processing (Fig 1). For unknown reasons, some samples did not lead to successful proliferative hASCs. Mesenchymal stem cluster differentiation markers CD73, CD90 and CD105 showed positivity in hASCs through flow cytometry assessment. All proliferating cell lines, including controls and cases, developed similarly (Fig 2, upper and lower panels, respectively), leading to elongation and branching shapes during the differentiation stages (from left to right in all cases). hASCs also enabled neurosphere formation after 2–4 days of induction with neurosphere media (Fig 1 middle right panel). As expected, bIII tubulin neuronal marker staining in undifferentiated hASCs did not lead to a detectable signal (Fig 1 bottom left panel), whereas the signal was present in hASC-exposed neurobasal differentiation media after 20 days of differentiation (Fig 1, bottom middle panel). Other neuronal markers were also tested, such as tyrosine hydroxylase, microtubule-associated-protein-2 (MAP-2) and dopamine transporter, leading to inconclusive results due to high background or unspecific fluorescence detection. Neurosphere immunostaining options were limited due to the tridimensional structure of the aggregates, although cytoplasmic cell and mitochondrial structural networks could be determined by β actin and TOM20, respectively (Fig 1, bottom right panel).

### hASC and NhASC proliferation capacity

**Fig 3:**
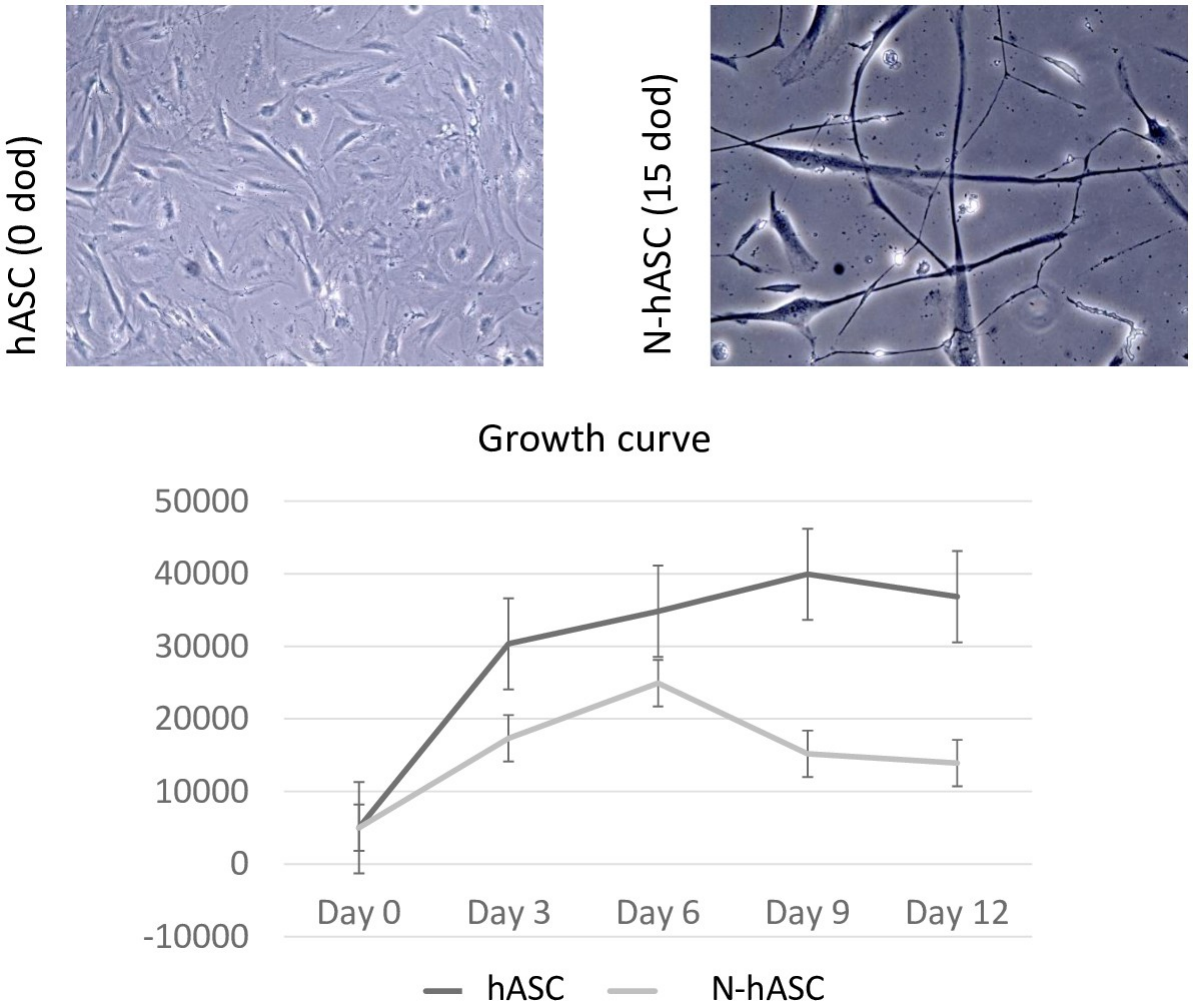
Representative images and growth curves of control hASCs and NhASCs. Upper panels, representative images of hASCs (left panel) and NhASCs (right panel) exposed to either standard culture media or neurobasal differentiation media, respectively, after 15 days of development. NhASCs presented lower confluence at 15 days of development through exposure to neurobasal media differentiation than hASCs on the same day of development under standard culture conditions. At the bottom, a standard growth curve conducted from 0 to 12 days suggests a lower proliferative capacity of NhASCs than of hASCs.

NhASCs showed lower proliferative capacity than undifferentiated hASCs, suggesting postmitotic neuronal-like features with less growth capacity (Fig 3). All proliferating controls and cases presented the same growth capacities in both basal and differentiating conditions.

### Neuronal differentiation protein markers

**Fig 4:**
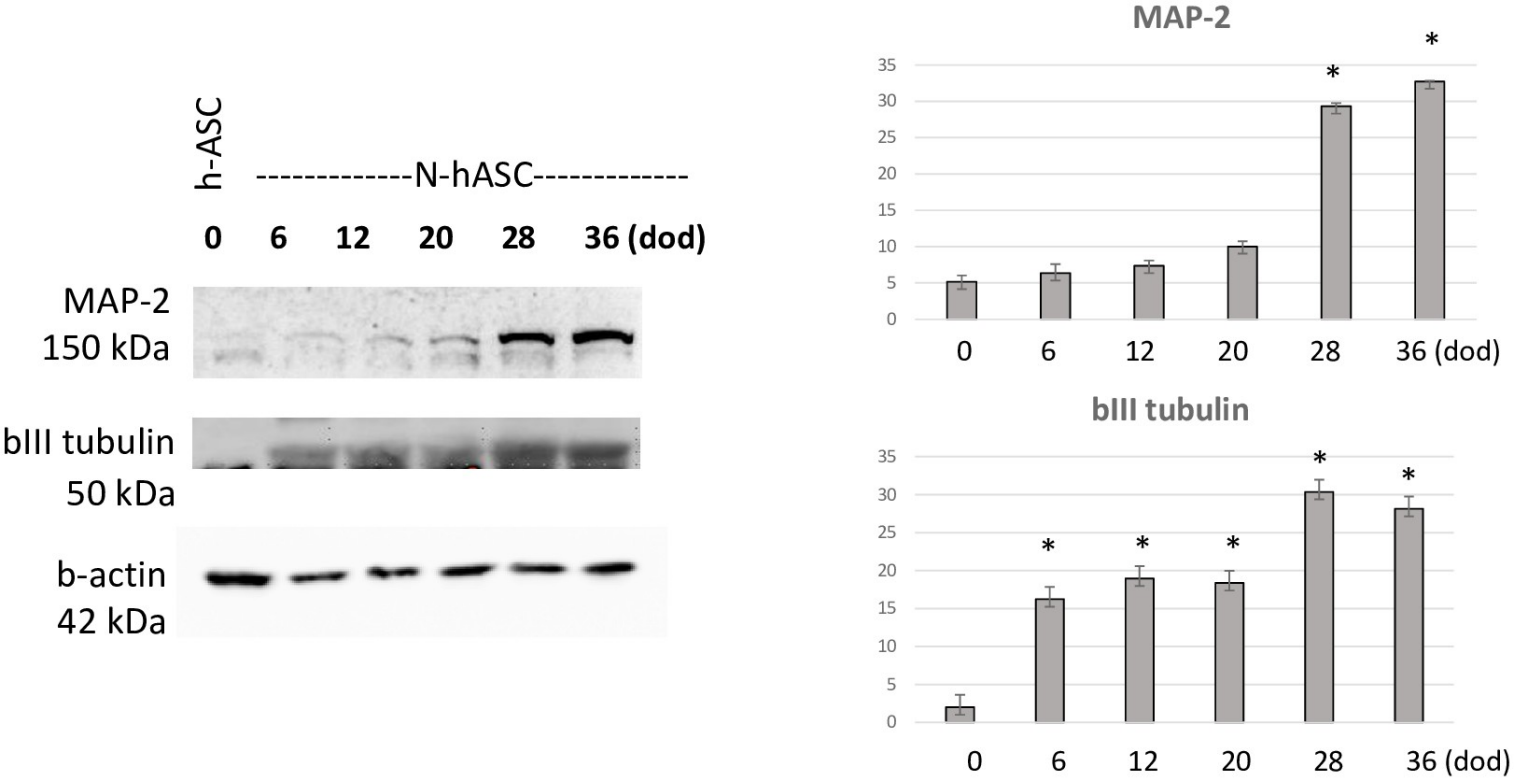
Representative blots and quantification of neuronal markers. Left panels, representative blots of MAP-2 and bIII tubulin neuronal marker protein expression in hASCs and NhASCs upon neuronal induction with neurobasal differentiation media over time, from 0 to 36 days of differentiation. From top to bottom, blots show signals of specific bands for neuronal MAP-2 and bIII tubulin and housekeeping b-actin (indicating similar protein loading in all samples). Right panels, densitometric quantification of MAP-2 neuronal marker protein expression upon neuronal induction (p<0.05 in undifferentiated hASCs at 0 days vs. 28 and 36 days of differentiation); and quantification of bIII tubulin neuronal marker protein expression upon neuronal induction (p<0.05 in hASCs at 0 days vs. 6, 28 and 36 days of development). AU, arbitrary units; Dod, day of differentiation; hASCs, human adipose stem cells; NhASCs, neuronal transdifferentiated human adipose stem cells.

Immunodetection of different neuronal markers was conducted. The dopaminergic markers tyrosine hydroxylase and dopamine transporter DAT did not lead to conclusive results due to the absence of signals in all the samples. In contrast, the microtubule-associated protein MAP-2 marker of mature nonproliferative neuronal cells led to positive staining, specifically in the last differentiation period, similar to bIII tubulin, an early biomarker of neural cell differentiation from multipotent progenitors [26], which led to a significant increase from 6 days of differentiation (Fig 4).

### Oxidative capacity of hASC control and pathological lines

#### High-resolution respirometry through Oroboros and SeahorseX24

**Fig 5:**
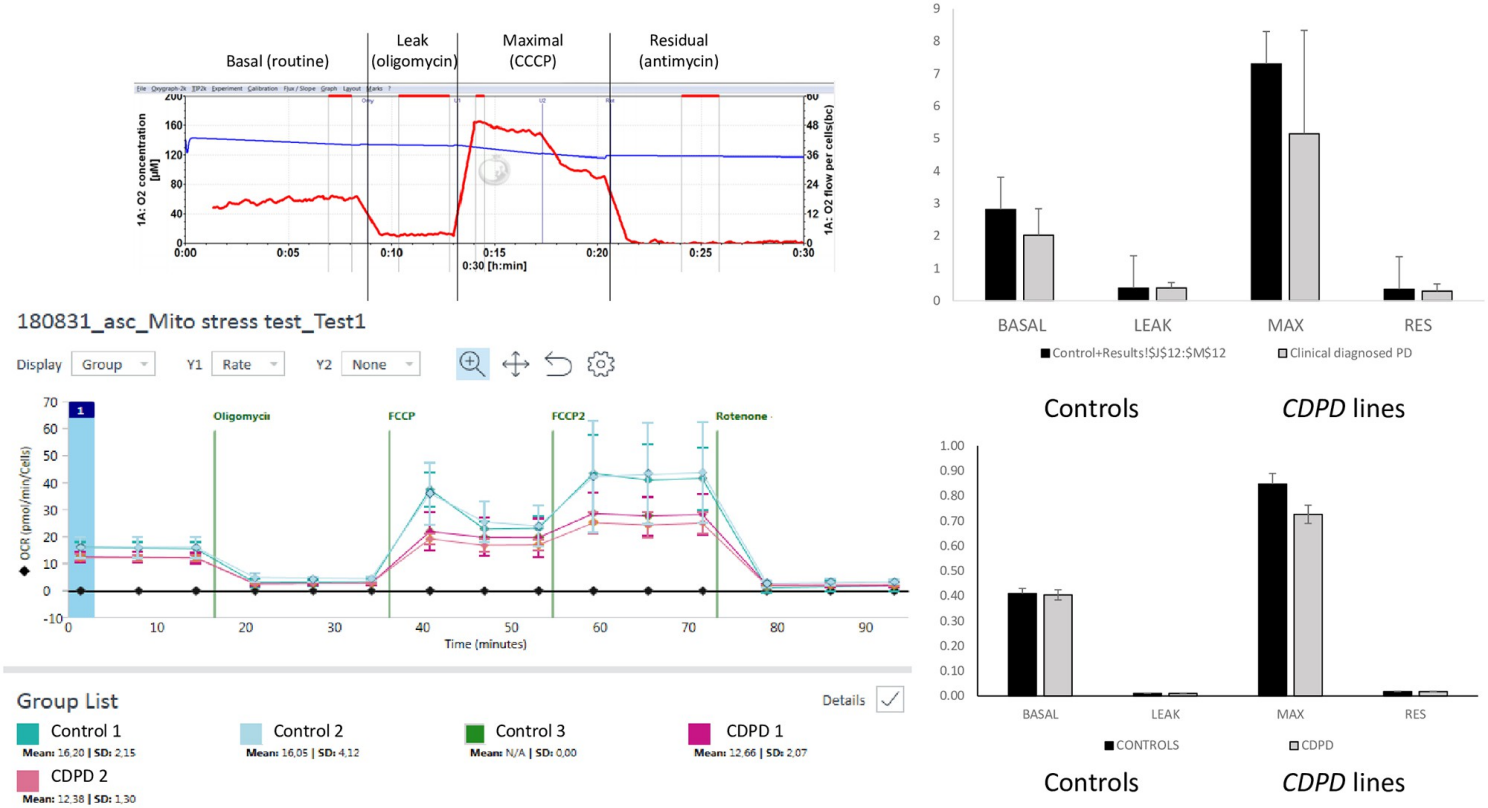
Bioenergetics profile through high-resolution respirometry. Upper graph, representative respiration OXPHOS slope states by Oroboros 2k-Oxygraph. Substrate and coupling states are shown. The blue curve indicates the O2 concentration in the O2k chamber, and oxygen fluxes per chamber volume are depicted (red curve) for the O2k chamber operated simultaneously. The upper horizontal bar denotes the respiratory states (routine, OXPHOS, leak and electron transport system states). Second graph, respirometry assessment and quantification of healthy control hASCs vs. *CDPD* hASCs. Basal or routine respiration of intact cells without any substrates or inhibitors, proton leakage after oligomycin inhibitor of ATP synthase or complex V, maximal respiratory capacity after CCCP uncoupler addition and final residual respiration after antimycin inhibition of the mitochondrial respiratory chain (specific inhibition of complex III). Basal oxidative respiration of intact cells tended to decrease respiration levels in the *CDPD* cases (p = 0.1). Third graph, representative respiration slope states by Seahorse X24 Agilent technology. Substrate and coupling states are shown in triplicate simultaneously through the 24-well system. Bottom graph, respirometry assessment and quantification of healthy control hASCs vs. *CDPD* hASCs. The same profile of basal respiration, proton leakage after oligomycin inhibition, maximal respiratory capacity after FCCP uncoupler titration and final residual respiration after rotenone-antimycin inhibition of the mitochondrial respiratory chain (specific inhibition of complexes I and III, respectively) is shown. Basal Respiration was calculated as (Basal-Rote/AA), Maximal Respiration was calculated as (FCCP-Rote/AA). For both Oroboros and Seahorse technologies, trends toward lower maximal respiration capacity were observed in PD hASCs. hASC, human adipose stem cells, *CDPD*, clinically diagnosed Parkinson’s disease; Max, maximal; Res, residual.

Mitochondrial energy metabolism can be comprehensively tested through Oroboros technology in real time by titrating various substrates, uncouplers, inhibitors, and other substances during the experiment. First, the oxidative profile of control hASCs was assessed and compared to that of PD hASCs. Basal respirometry, proton leakage after oligomycin addition, maximal respiratory capacity through uncoupler titration and residual respiratory activity after inhibition of the mitochondrial respiratory chain were determined (Fig 5). Nonsignificant trends toward lower basal respiratory performance in intact cells were observed in the PD hASCs compared to controls. To further confirm such observed trends in basal respiration of PD cases, further oxidative experimental assessments using 24-well Seahorse Agilent technology were conducted. Similar to previous findings, Seahorse data, including basal, leak, maximal and residual respiratory capacity, led to comparable results, confirming the lack of differences between controls and PD hASCs and the suitability of hASCs to undergo high-resolution respirometry analysis (Fig 5).

Despite the lack of significant differences in respirometry measurements, trends toward suboptimal maximal respiration capacity in PD hASCs were confirmed in both oxygraphs compared with the healthy control hASC lines.

**Fig 6:**
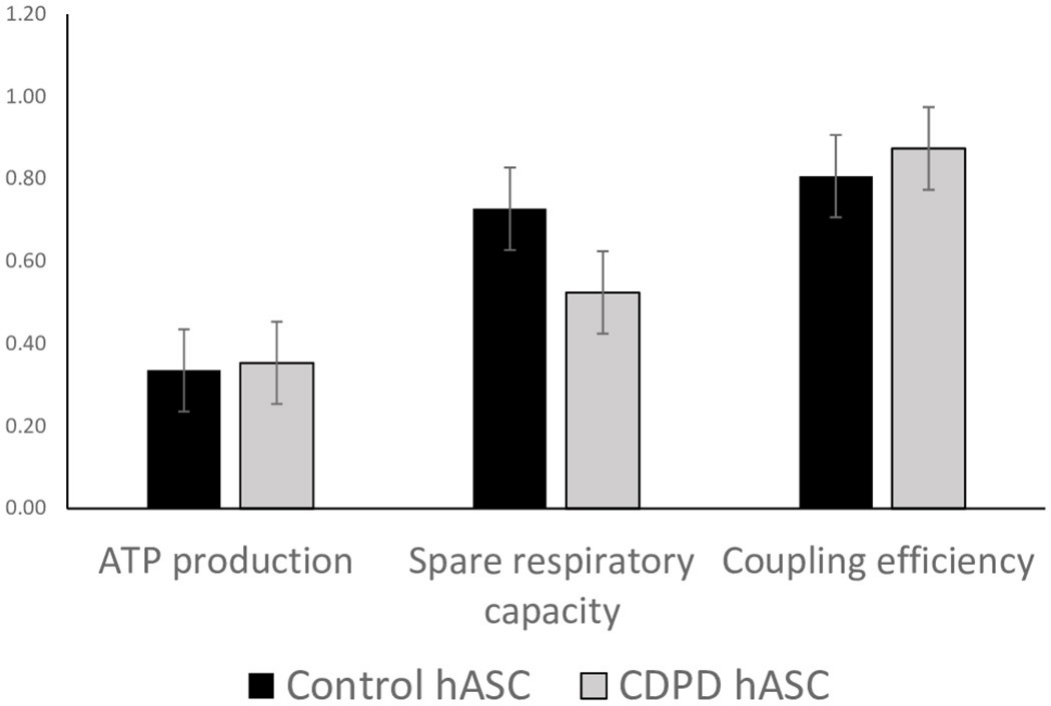
Oxidative parameters: ATP production, spare respiratory capacity and coupling efficiency of the mitochondrial respiratory chain. ATP production was calculated as (basal-H+ leak), spare respiratory capacity % was calculated as (maximal/basal), and coupling efficiency was calculated as (ATPprod/basal). No significant differences were found between the control and *CDPD* hASC lines. AU, arbitrary units; hASC, human adipose stem cells; *CDPD*, clinically diagnosed Parkinson’s disease.

Further respirometry parameters were also explored to confirm that the oxidative state of the control and PD hASCs led to nonsignificant differences between the groups (Fig 6).

## Discussion

The present study reports the characterization of hASCs considering their ability to undergo neuronal-like transdifferentiation and their bioenergetic fingerprint. Two neuronal-like transdifferentiation procedures were adequately performed by cultured hASCs derived from forearm human healthy controls and PD patients. First, neuronal-like features were found in cultured NhASCs under exposure to neurobasal differentiation media. Morphologically, branching and elongation of NhASCs were observed upon chemical induction, as already depicted in the literature [27]. On the other hand, the expression of early and mature neuronal protein markers, bIII tubulin and MAP-2, respectively, was observed in the NhASC model and was stable for several differentiation days. An increase in bIII expression was achieved at 6 days of neuronal differentiation, whereas an increase in MAP-2 was reached at 28 days of neuronal differentiation. The period needed to detect the increase in such protein expression markers varied as expected, considering that bIII tubulin is an indicator of early neuronal models [26] and MAP-2 is a biomarker for mature neuronal models [28]. Second, adequate neurosphere generation was observed under exposure to neurosphere induction media, as previously reported [20, 29]. Neurosphere generation was conducted to test another neuronal transdifferentiation potential capacity of the hASC model in this study, leading to proper neurosphere generation. In addition, the characterization of the hASCs included the assessment of the oxidative profile to decipher the bioenergetic fingerprint of the model, leading to similar trends between healthy controls and PD patients. To date, this is in contrast with suboptimal bioenergetics reported in other PD models [1]. However, comparable basal respiration has also been described in PD cybrid models compared to controls [30], and similar to this study, nonsignificant trends have also been described in hASCs in the literature [18]. Despite the lack of significant differences in respirometry measurements, trends toward suboptimal maximal respiration capacity in PD hASCs were confirmed in both oxygraphs compared with the healthy control hASC lines.

Limited sample inclusion is probably the reason for comparable preserved results in the bioenergetic assessment of the hASC model, but this is not precluded by the relevant finding related to positive neuronal protein expression upon induction of the model.

To gain knowledge of the biological mechanisms of hASCs is an urgent need, since they represent promising treatment tools for various disorders. Recently, hASCs have been described to present a protector effect in motor neurons and to be associated with reduce glial activation in both in vitro and in vivo models of ALS [31]. The effectiveness and mechanisms of adipose-derived stem cell therapy in animal PD models have already been depicted in the literature. Transplantation of ASCs represents a therapeutic approach with long-lasting effects in PD animal models. Following the transplantation of ASCs, tyrosine hydroxylase-positive neurons (dopaminergic neurons) have been described to recover in the lesion [32]. The potential mechanisms of ASCs involve neuroprotective effects and neurogenesis, and the standardized induction of the neural form of grafted ASCs can lead to future approaches in the clinical practice. Unfortunately, in our hASC transdifferentiation model, we could not detect tyrosine hydroxylase positivity or other dopamine-related markers, such as DAT. Standardization of optimal transdifferentiation procedures is an urgent need. Optimal conditions for hASC procedures are still a matter of debate in a field that is gaining strength and remains to be explored.

For instance, in a recent study, hASC under hypoxic culture conditions was described to improve neuronal differentiation and nerve repair [33]. The transdifferentiation ability of human bone marrow-derived stem cells to neurons upon chemical induction was previously described [34, 35]. As mesenchymal stem cells share many biological features with ASCs, this protocol has been adapted for neural ASC differentiation with different modifications. This well-known method has been experimentally revisited several times, as the stage-specific incidence of ASC differentiation needs to be validated [27]. Although the optimal procedure for hASC transdifferentiation to neural lineages needs to be better defined, representing a limitation for this and other similar studies, the protocol for neuronal ASC induction herein has been previously reported [36].

Further precision of the protocols would identify a promising source for neural tissue replacement and is likely to occur imminently. However, the main limitation of this study was the limited sample size regarding both sources of the sample, which probably hampered successful proliferative cultures (the low amount of tissue led to nonproliferative cell cultures in some cases) and regarding the number of subjects included in the study, which may mask differential bioenergetics profiles between controls and pathological lines (the low number of subjects was likely to promote loss of statistical power). Indeed, the forearm origin of our samples led to a limited source of biological material, but suitability of the use of adipose tissue should also be considered an advantage for the present model. In fact, among the various types of mesenchymal stem cells, hASCs are relatively easy to obtain through subcutaneous fat aspiration, liposuction, other surgery interventions and methods [5]. The relatively high vascularization occurring in the forearm is an advantageous feature to optimize adipose stem cells proliferation. Compared with harvesting bone marrow stem cells, this process is less invasive and less controversial than the use of embryonic stem cells. In addition, transdifferentiation allows direct differentiation properties as an advantage that is absent in other cell models, such as induced pluripotent stem cells (IPSCs) [37], although potential neurodevelopmental features may not be detected in this model compared with IPSCs.

In summary, we confirmed the capability of transdifferentiation to the neuronal-like profile of hASCs derived from the forearms of human subjects and characterized the bioenergetic oxidative profile of hASCs, which did not lead to significant differential respiration performances in healthy controls and PD patients. The limited sample size could explain the lack of statistical significance, and further studies following that direction are needed.

## Conclusions

Human adipose stem cells derived from the forearm of the study subjects allow for neuronal transdifferentiation and bioenergetics assessment. They may therefore represent a suitable tool to model and investigate molecular features related to Parkinson’s disease and other neurodegenerative processes and to test potential pharmacological strategies. A main limitation of the study is the limited sample size that could also explain the lack of statistical significance, and further studies following that direction are needed.

## Supporting information

S1 Data (XLSX)

S1 Raw images (PDF)
