# Supplementary figures and images for: Neuronal induction and bioenergetics characterization of human forearm adipose stem cells from Parkinson’s disease patients and healthy controls
Source: PLoS One. 2022 Mar 15;17(3):e0265256. doi: 10.1371/journal.pone.0265256 (PMC8923468; doi:10.1371/journal.pone.0265256)

BIII tubulin

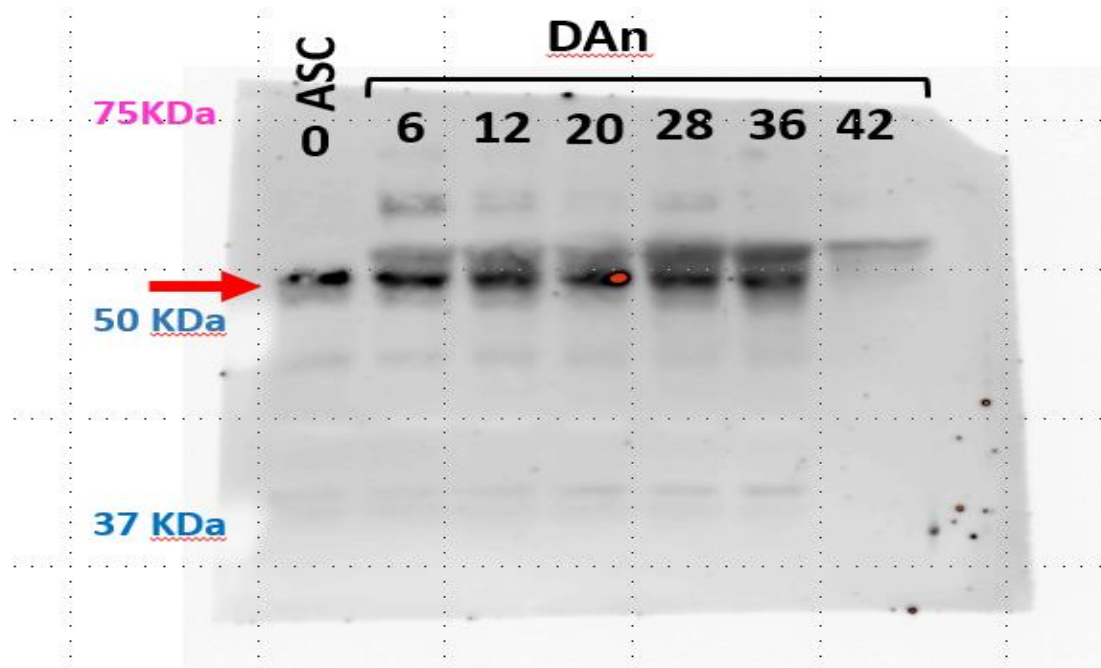

MAP2

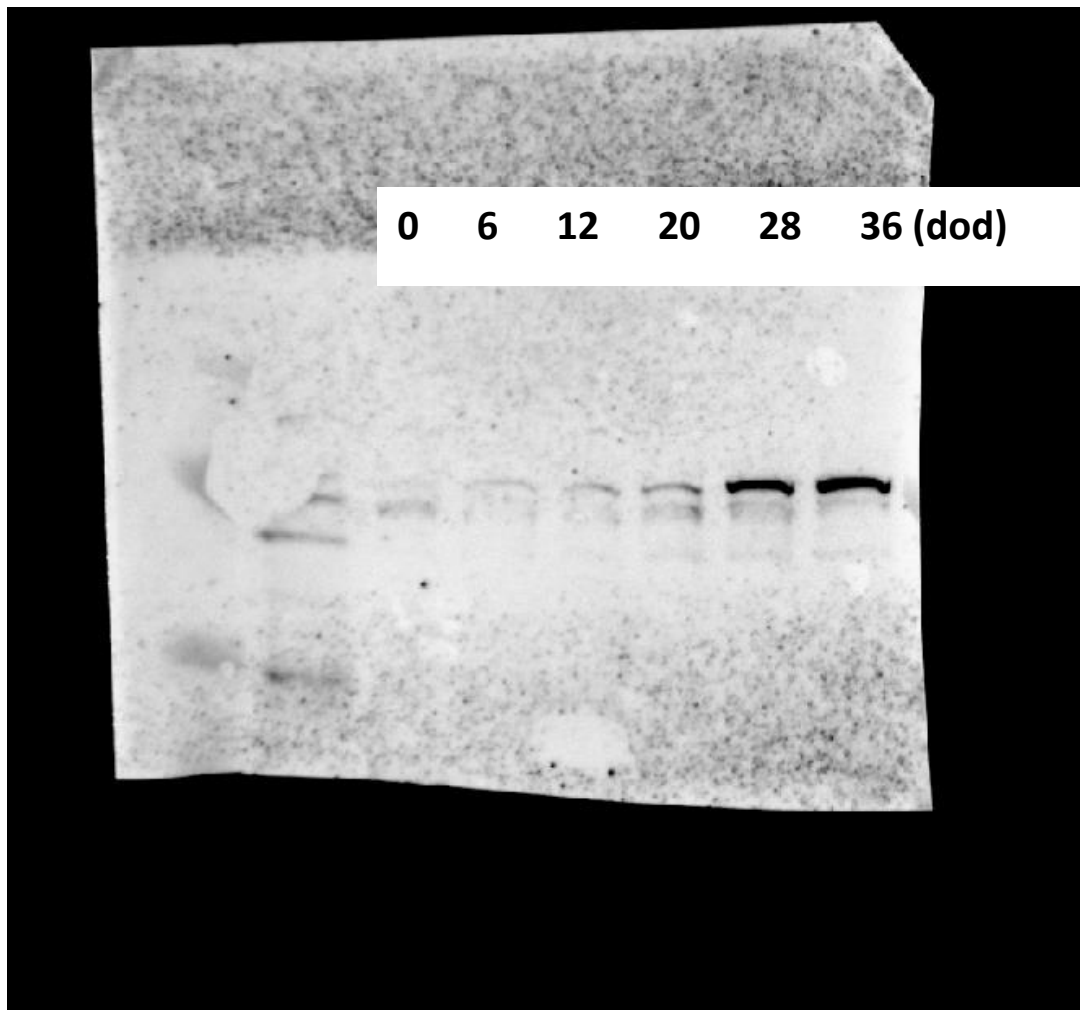

B actin

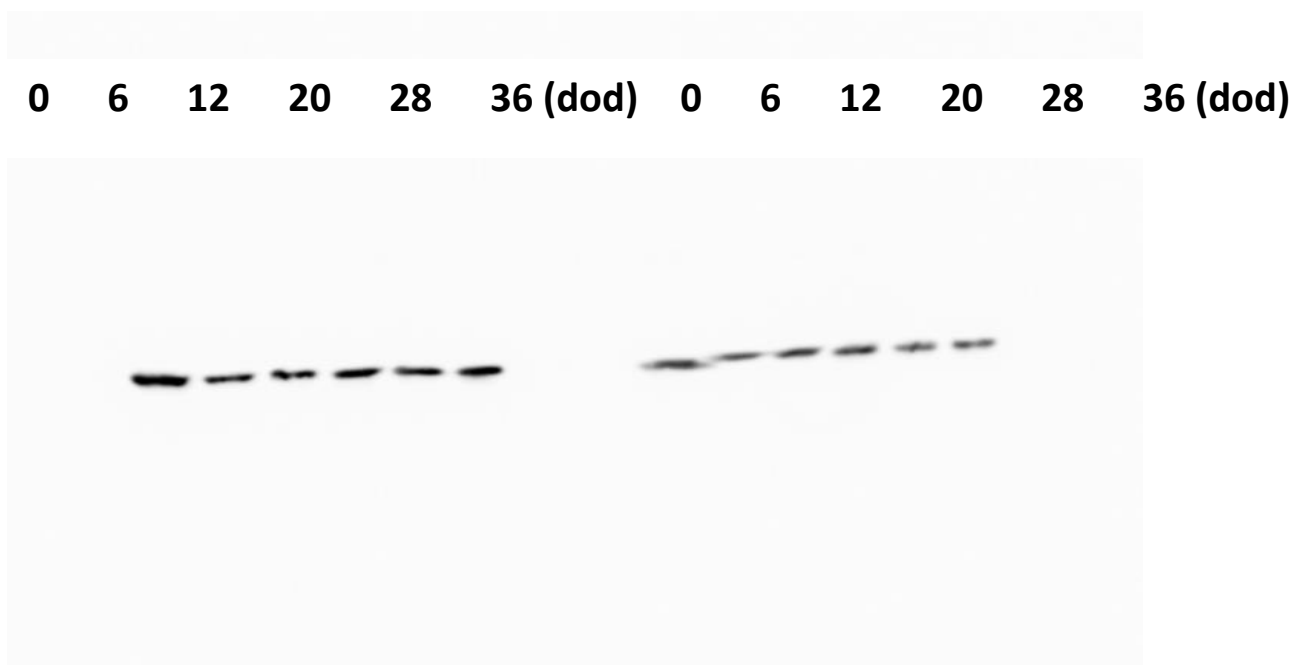

Supplement: S1 Raw images — (PDF) [file pone.0265256.s002.pdf]
